# Association Between Efficacy of Immune Checkpoint Inhibitors and Sex: An Updated Meta-Analysis on 21 Trials and 12,675 Non-Small Cell Lung Cancer Patients

**DOI:** 10.3389/fonc.2021.627016

**Published:** 2021-08-26

**Authors:** Chongxiang Xue, Shuyue Zheng, Huijing Dong, Xingyu Lu, Xu Zhang, Jingyi Zhang, Jia Li, Huijuan Cui

**Affiliations:** ^1^China-Japan Friendship Clinical Medical College, Beijing University of Chinese Medicine, Beijing, China; ^2^Department of Integrative Oncology, China-Japan Friendship Hospital, Beijing, China; ^3^Department of Clinical Oncology, Li Ka Shing Faculty of Medicine, The University of Hong Kong, Hong Kong, Hong Kong, SAR China

**Keywords:** immune checkpoint inhibitors, non-small cell lung cancer (NSCLC), different sexes, meta-analysis, patients’ selection

## Abstract

**Background:**

Mounting randomized clinical trials have proved that immune checkpoint inhibitors (ICIs) achieved better overall survival (OS) and progression-free survival (PFS) than chemotherapy drugs for advanced non-small cell lung cancer (NSCLC) patients. However, some literatures have indicated that different sexes might not have equal immune response. Also, no agreement reached on the issue whether therapeutic benefit of ICIs is related to sex.

**Objectives:**

To explore the association between efficacy of ICIs for NSCLC patients and their sexes and summarize overall treatment-related adverse events (TRAEs) in an exploratory manner.

**Methods:**

We performed this systematic review and meta-analysis of all potentially relevant studies retrieved from PubMed, EMBASE, and the Cochrane Library until June 2021, for eligible randomized controlled trials (RCTs) comparing immunotherapy with chemotherapy in advanced NSCLC patients. Literature screening, summary data extraction was performed independently and in duplicate. The pooled hazard ratio (HR) and 95% confidence interval (CI) of OS, PFS and TRAEs were calculated, applying STATA software and random-effects models. This study was registered in international prospective register of systematic reviews (PROSPERO), number CRD42020210797.

**Results:**

Twenty-one trials involving 12,675 NSCLC patients were included. For patients with advanced NSCLC, ICIs significantly prolonged the OS (males: HR 0.73, 95%CI 0.67-0.79; females: HR 0.73, 95%CI 0.61-0.85) and PFS (males: HR 0.62, 95%CI 0.55-0.70; females: HR 0.68, 95%CI 0.55-0.81) *versus* chemotherapy. Overall, there was no statistical difference between their sexes (OS: P = 0.97; PFS: P = 0.43), respectively. Owing to insufficient TRAEs data of different sexes, we only found immunotherapy for NSCLC patients had more all-grades (RR 0.88; 95%CI 0.82-0.95) and 3-5 grades (RR 0.60; 95%CI 0.47-0.75) AEs compared with chemotherapy.

**Conclusion:**

Our findings indicated that the interaction between immunotherapy efficacy and different sexes was equally evident. Overall, patients with NSCLC could obtain more benefits from ICIs than chemotherapy regimen regardless of their sexes.

**Systematic Review Registration:**

PROSPERO (https://www.crd.york.ac.uk/prospero/), identifier CRD42020210797.

## Introduction

Lung cancer is one of the most common thoracic diseases, and NSCLC accounts for approximately 85% of total histological subtypes (1). It has reached epidemic proportions and always been the leading cause of cancer related deaths worldwide (2). According to global cancer statistics, frequency of new diagnosis reaches to 22.5 per 100,000, with death rates 18.6 per 100,000 (3, 4). In spite of tremendous advances in local and systemic therapies, cure rates of lung cancer were still slowly increased over the last decades (5). In recent years, the advent of immunotherapy has brought about a shift in the landscape of NSCLC treatment (6–8). More clinical trials have demonstrated that ICIs have a higher OS or PFS than chemotherapy for NSCLC patients (9).

Sex correlations seem to exist in lung cancer for the fact that males have a higher incidence (31.5% *vs* 14.6%) and mortality (27.1% *vs* 11.2%) than females (10). However, we still have no clear ideas of efficacy of ICIs in different sexes. Previously, Wallis and colleagues (11) updated a meta-analysis and found that there was no statistical significance between efficacy of ICIs and sex in the treatment of various advanced cancers. However, heterogeneity exists, and different varieties of tumors do not have equal outcomes for ICIs (12). As a result, Wang and colleagues (13) had drawn that controversial conclusion that males obtain more beneficial outcomes from ICIs than females in NSCLC in their subgroup analysis.

Now that these previous studies have not come to consistent findings on this issue, a comprehensive updated meta-analysis is necessary to yield more information. What’s more, we noticed that Wang and colleagues (13) did not perform a test of interaction to compare the difference of outcomes data between males and females. Statistical data, including hazard ratio (HR) and P value, were insufficient to support its final conclusion. Also, several comprehensive and worthy clinical trials had updated outcomes data, which might influence conclusions in this literature review. Consequently, we aim to conduct an analysis of 12,675 patients to compare efficacy and safety of ICIs in NSCLC and patients’ sex.

## Materials and Methods

### Literature Search and Selection Criteria

Our study was registered in PROSPERO, number CRD42020210797. And this systematic review and meta-analysis complied with the Preferred Reporting Items for Systematic Reviews and Meta-Analyses (PRISMA) Statement (14).

We searched for all potentially relevant studies retrieved from PubMed, EMBASE, and the Cochrane Library until June 2021, for eligible phase II or III RCTs comparing immunotherapy with chemotherapy in stage IIIB or IV NSCLC patients. And we searched keywords and Medical Subject Headings (MeSH) terms pertinent to the intervention of interest. Articles published in non-English-language were excluded. More details about procedures and methods were reported in the appendix.

### Data Extraction

The data was extracted by two authors (CXC and HJD) independently. The following information was extracted from the trials: first author, year of publication, histology of lung cancer, therapeutic line, trial phase, immunotherapy targets, number of patients, intervention arms, control arms, median follow-up time, PFS/OS hazard ratio (HR) of males and females, all grades and 3-5 grades TRAEs. The third author (HJC) assessed the data and resolved the disagreement.

### Assessment of Study Quality and Publication Bias

The Cochrane collaboration tool (15) was applied to assess studies for methodological quality. There are seven aspects, including selection bias, allocation concealment, performance bias, detection bias, attrition bias, reporting bias and other bias. All the included clinical studies have been registered. All assessments were independently verified by two authors (CXX and HJD). Any disagreements were resolved through discussion with the third author (HJC). Potential publication bias among the main outcome was assessed by Begg’s test.

### Statistical Analysis

The STATA software (version 14.0) was used for statistical analyses and generation of the forest plots. HR and 95%CI were used as effect sizes. The pooled estimates were considered statistically significant if the 95% CI did not include 1.0, with a P value of <0.05 (two-sided).

Statistical heterogeneity across studies was assessed using the I^2^statistic and forest plots. An I^2^ value of <50% indicated a low heterogeneity (16). In this analysis, the null hypothesis that the studies were homogenous would be rejected if P for heterogeneity was less than 0.10 or I^2^ > 50%. Owing to heterogeneity inherent in clinical data, random-effects model was applied to calculate the summary estimates.

We used the inverse variance method, assuming that the studies included in the meta-analysis had the same quantity. We made calculations using log HR, comparing two estimates from the same patients derived from separate analyses with test of interaction. Moreover, we assessed whether the variations differed from the null using the χ2 test.

We explored the heterogeneity *via* subgroup analysis using the following classification variables: target class of ICIs (PD-1, PD-L1, CTLA-4, or combination), line of therapy (first line, or after first line), study methodology (IO+chemo *vs* chemo, IO *vs* chemo, IO+IO *vs* chemo), and different pathological types (NSCLC, squamous NSCLC, non-squamous NSCLC). We also use a test of interaction to compare OS/PFS data in male and female groups derived from separate analyses. Outcomes of interest provided by over two studies are pooled and presented.

## Result

### Search Results and Patients’ Characteristics

**Figure 1:**
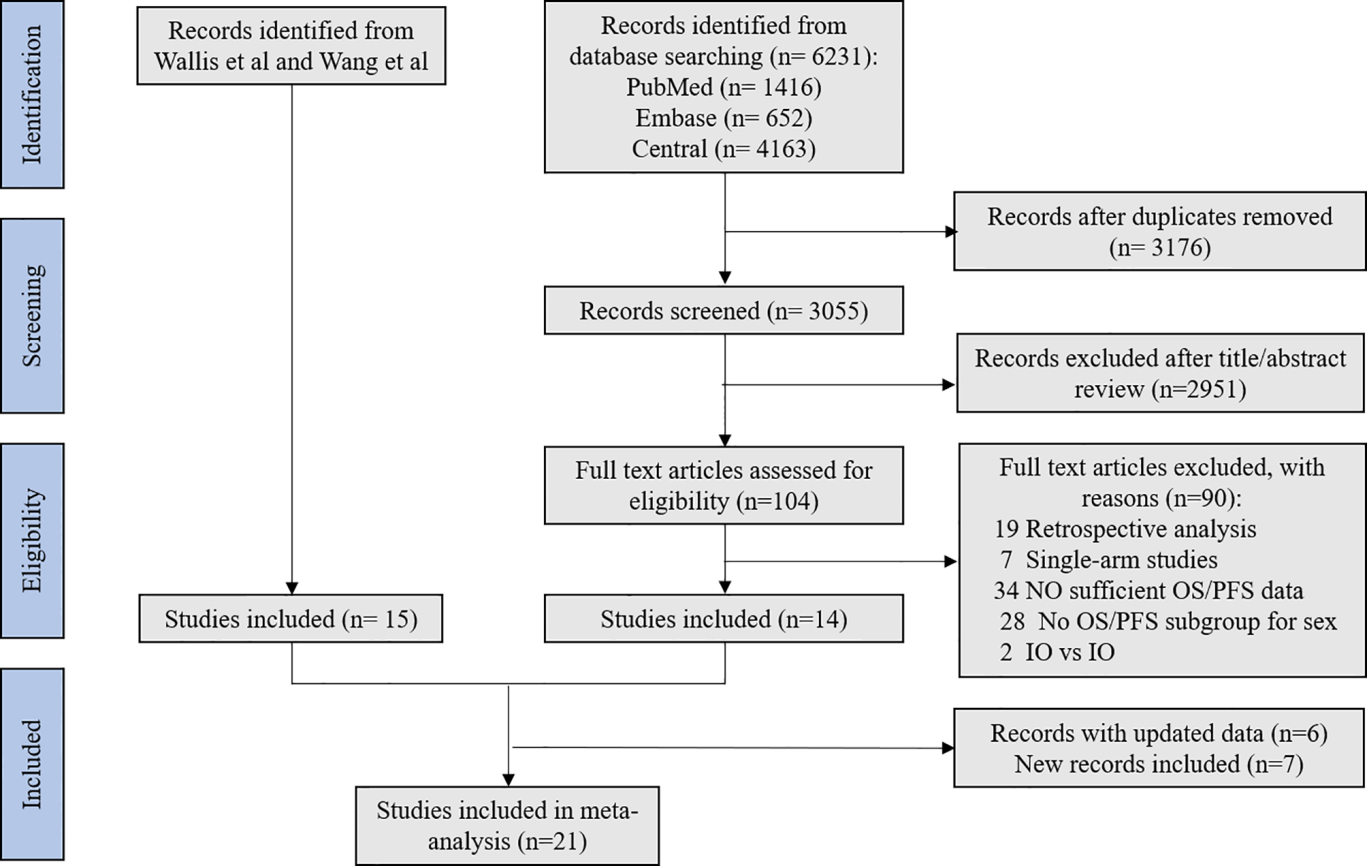
Flow chart for study selection.

On the basis of the initial search strategy, we identified 6231 potentially relevant articles, of which 3176 were duplicates. After eligibility screening of the titles and abstracts, 21 identified trials (17–38) were deemed eligible for inclusion. Finally, 12,675 patients were enrolled. **Figure 1** depicts the search process.

Additionally, baseline characteristics of these trials were summarized in **Table S1**. All the trials evaluated efficacy of ICIs for males and females, including 5 with Nivolumab, 6 with pembrolizumab, 5 with Atezolizumab, 3 with Ipilimumab, 1 with Avelumab, 1 with Sintilimab, 1 with Cemiplimab, and 1 with Tislelizumab. All the studies were well designed phase II or III RCTs. Ten trials investigated PD-1 blocking agents, eight trials investigated PD-L1 blocking agents, two trials investigated CTLA‐4+ PD-1 blocking agents, and only one trial investigated CTLA‐4 blocking agents.

### Quality of the Included Studies and Publication Bias

**Figure 2:**
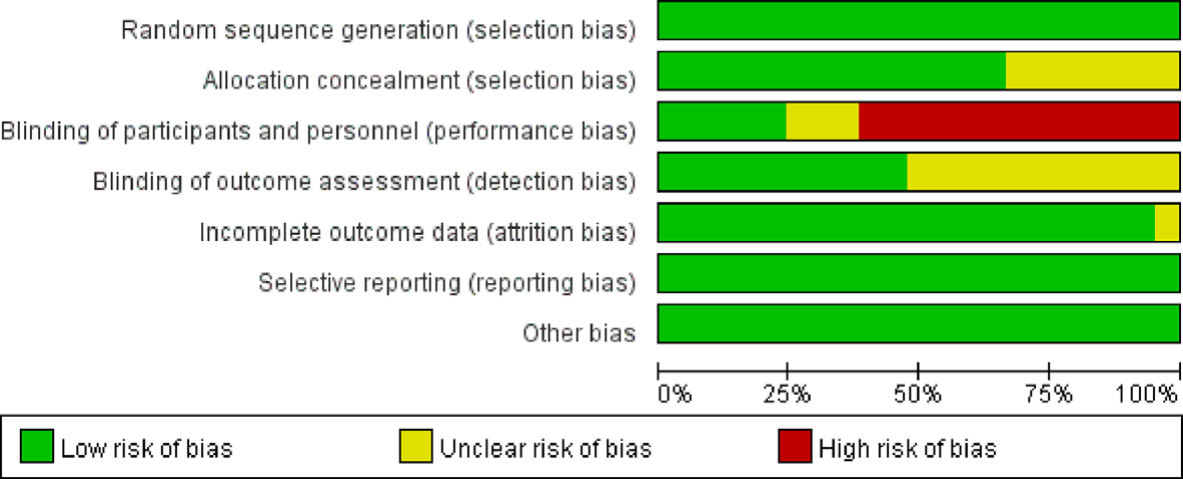
Risk of bias graph assessed by the Cochrane collaboration tool in Revman 5.3.

**Figure 3:**
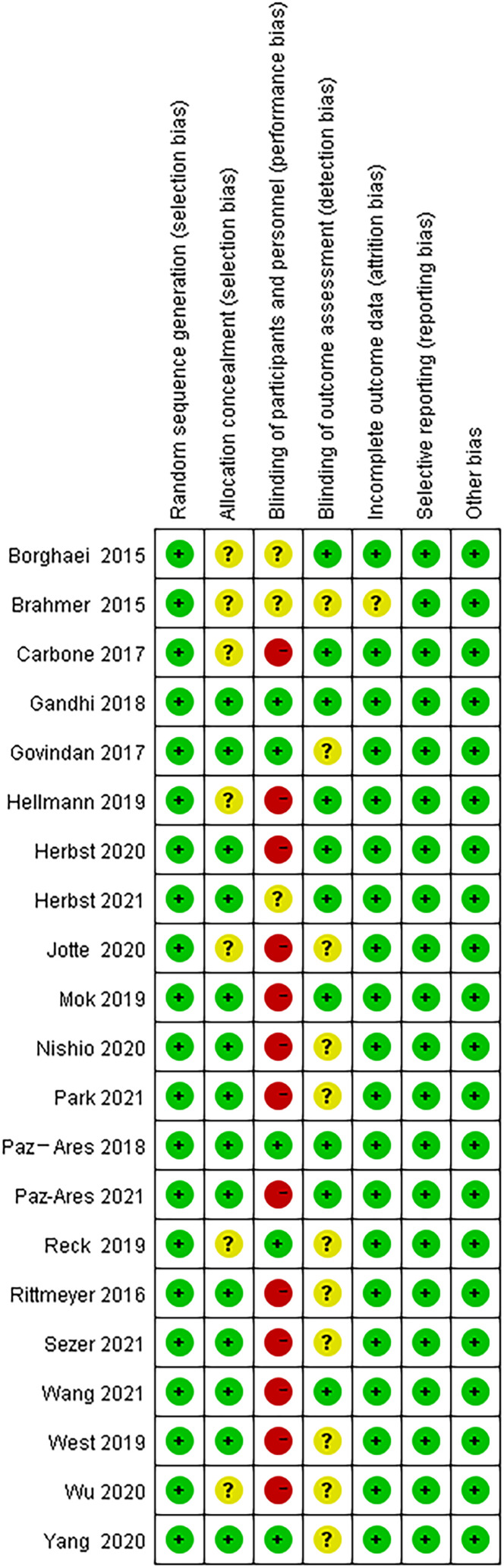
Risk of bias summary assessed by the Cochrane collaboration tool in Revman 5.3.

**Figure 4:**
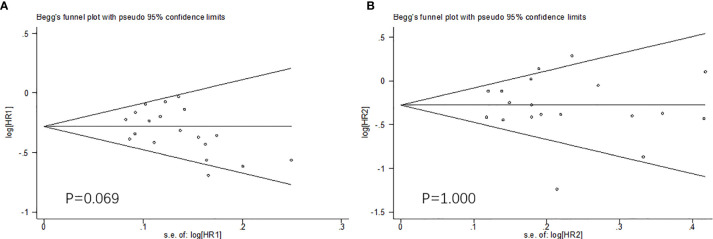
The Begg’s test and funnel plots (OS: **A.** males; **B.** females). No publication bias were observed.

Because of the difficulty of masking, some of the included studies showed high risks and unknown risks. All of them had comparatively comprehensive information of outcomes data. The assessment of bias was detailed in **Figures 2** and **3**. No publication bias for OS were observed in those studies (P = 0.069 and 1.000) by Begg’s test and the funnel plots were shown in **Figure 4**.

### Outcomes

**Figure 5:**
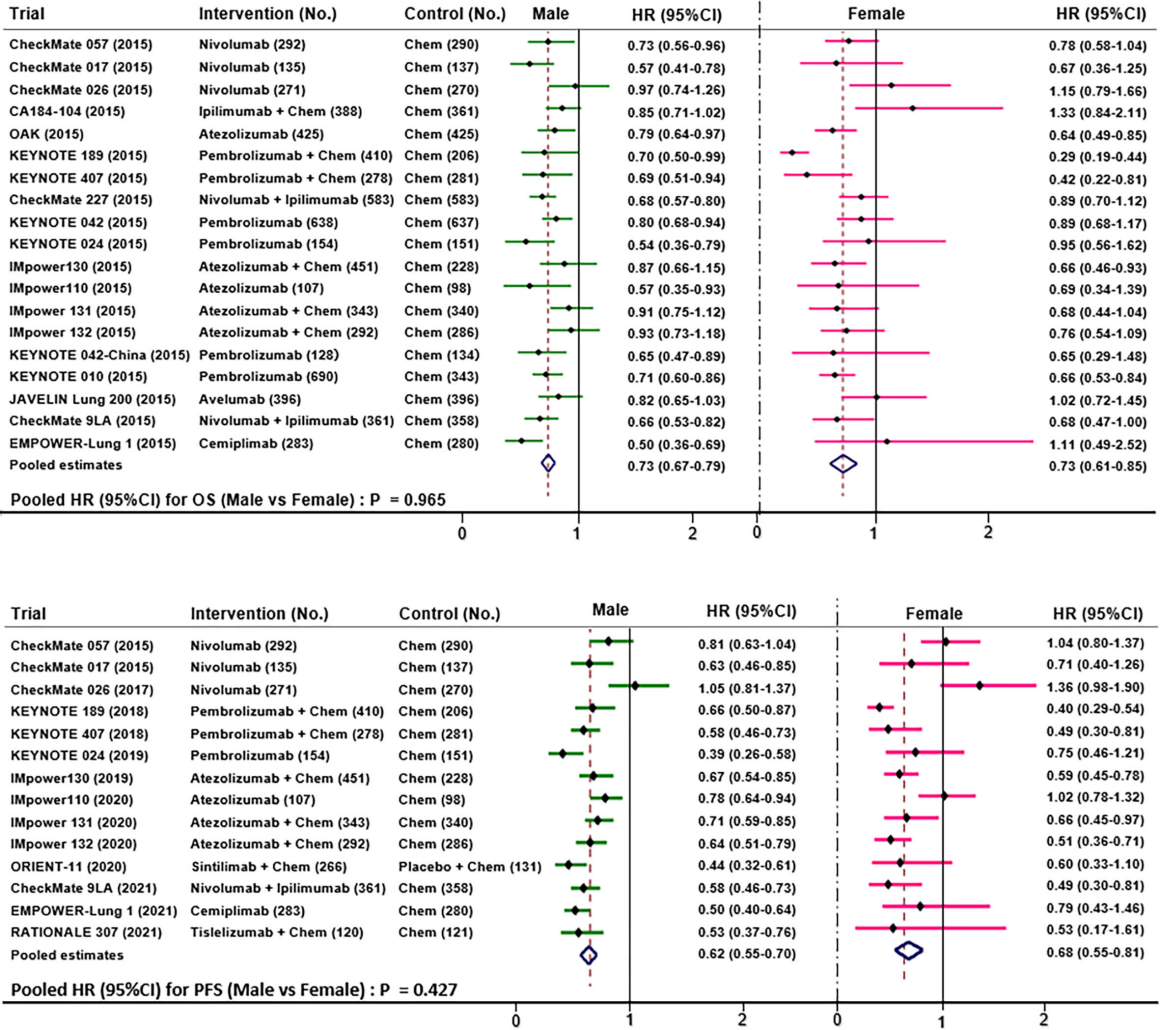
Funnel plots depicting pooled OS **(A)** and PFS **(B)** data.

Nineteen trials compared OS data according to NSCLC patients’ sex. This meta-analysis showed (**Figure 5A**) that the pooled OS HR was 0.73 (95%CI 0.67-0.79) for males and 0.73 (95%CI 0.61-0.85) for females when treated with ICIs *versus* chemotherapy. The clinical benefit was not statistically significant for OS results between males and females (HR 1.00; 95% CI 0.92-1.08; P=0.97).

Fourteen trials compared PFS data according to NSCLC patients’ sex. This meta-analysis showed (**Figure 5B**) that the pooled PFS HR was 0.62 (95%CI 0.55-0.70) for males and 0.68 (95%CI 0.55-0.81) for females when treated with ICIs *versus* chemotherapy. Notably, the overall result of PFS manifested that males seemed not to benefit more from ICIs than females (HR 0.96; 95% CI 0.87-1.05; P=0.43).

### Subgroup Analysis

In general, males with NSCLC obtained similar OS and PFS benefits with females when treated with immunotherapy regardless of any subgroups (**Table 1** and **Figures S1**, **2**).

**Table 1:** Subgroup analysis of pooled OS HR and PFS HR.

Variable	Study No. (%)	Participants, No.		Pooled HR (95%CI) for OS			Study No. (%)	Participants, No.		Pooled HR (95%CI) for PFS		
		Men	Women	Men	Women	P value		Men	Women	Men	Women	P value
**Overall**	19	8286	3751	0.73 (0.67-0.79)	0.73 (0.61-0.85)	0.965	14	4852	2088	0.62 (0.55-0.70)	0.68 (0.55-0.81)	0.427
**Immune target**												
PD-L1	8	2872	1210	0.80 (0.74-0.87)	0.73 (0.63-0.83)	0.258	9	2864	1212	0.70 (0.63-0.77)	0.67 (0.48-0.87)	0.784
PD-1	8	2977	1494	0.66 (0.57-0.75)	0.68 (0.46-0.89)	0.916	4	1484	661	0.60 (0.49-0.70)	0.72 (0.51-0.94)	0.313
CTLA-4	1	635	114	0.85 (0.69-1.00)	1.33 (0.70-1.97)	0.112	-	-	-	-	-	
PD-1+CTLA-4	2	1282	603	0.67 (0.58-0.76)	0.80 (0.60-1.00)	0.236	1	504	215	0.58 (0.44-0.71)	0.49 (0.24-0.75)	0.593
**line of therapy**												
1	12	5297	2213	0.70 (0.63-0.78)	0.75 (0.55-0.95)	0.649	10	3384	1441	0.60 (0.51-0.69)	0.67 (0.50-0.84)	0.471
>1	7	2989	1538	0.77 (0.68-0.86)	0.71 (0.62-0.79)	0.307	4	1468	647	0.69 (0.61-0.77)	0.72 (0.47-0.96)	0.824
**study methodology**												
IO+chemo *vs* chemo	5	2270	1181	0.81 (0.74-0.88)	0.61 (0.43-0.80)	0.093	6	2157	899	0.65 (0.52-0.78)	0.63 (0.42-0.83)	0.877
IO *vs* chemo	10	4255	2095	0.69 (0.60-0.77)	0.78 (0.68-0.87)	0.158	8	2695	1189	0.61 (0.52-0.70)	0.72 (0.56-0.89)	0.238
IO+IO *vs* chemo	1	635	114	0.68 (0.56-0.80)	0.89 (0.68-1.10)	0.076	-	-	-	-	-	
**Pathological type**												
Squamous NSCLC	4	1855	408	0.76 (0.61-0.91)	0.69 (0.40-0.98)	0.702	4	1438	317	0.63 (0.55-0.70)	0.59 (0.43-0.75)	0.671
Non-squamous NSCLC	4	1466	989	0.80 (0.69-0.92)	0.61 (0.33-0.89)	0.290	4	1466	989	0.68 (0.60-0.76)	0.61 (0.40-0.81)	0.567
NSCLC	11	4965	2354	0.70 (0.63-0.77)	0.78 (0.68-0.88)	0.176	6	1948	782	0.60 (0.45-0.75)	0.82 (0.56-1.07)	0.138

Chemo, Chemotherapy; IO, Immunotherapy; NSCLC, Non-small cell lung cancer; PD-L1, Programmed cell death 1 ligand 1; PD-1, Programmed cell death protein 1; CTLA-4, Cytotoxic T - Lymphocyte Antigen 4.

### Adverse Events

**Figure 6:**
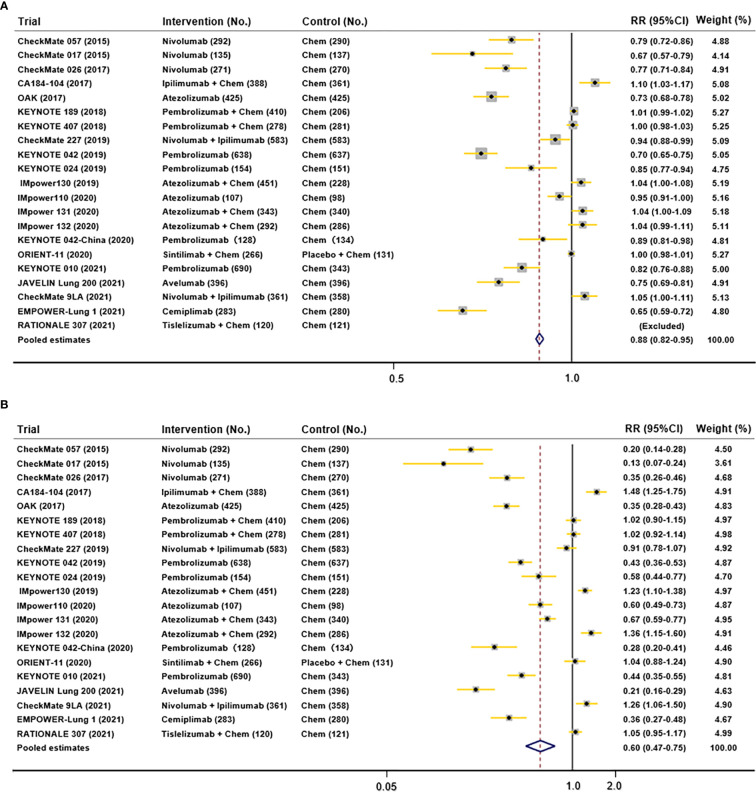
Funnel plots depicting pooled AEs data in included studies (**A.** all-grades TRAEs; **B.** 3-5 grades AEs).

All trials reported any grades of TRAEs and 3-5 grades of TRAEs for NSCLC patients. The pooled RRs of any-grade and grade 3–5 TRAEs% were 0.88 (95% CI 0.82-0.95), and 0.60 (95% CI 0.47-0.75). All reported AEs in those trials included did not demonstrate any subgroup analysis stratified by sex. As a result, a meta-analysis of AEs incidence according to sex was not feasible. Results of pooled AEs (**Figure 6**) showed that use of ICIs had more all-grades and 3-5 grades AEs compared with chemotherapy.

## Discussion

Immunotherapy drugs exert anti-tumor activity by inhibiting the immune escape caused by tumor cells, which is closely related to human immune system (39). PD-1/PD-L1 and CTLA-4 pathway are critical in tumor immune evasion and considered as attractive targets for therapeutic intervention (26). Monoclonal antibodies of PD-1/PD-L1 and CTLA-4 have proved to be promise and profit for lung cancer patients (40).

Previously, Wang and colleagues (13) drew the conclusion that males had better OS and PFS than females in NSCLC patients treated with immunotherapy by the comparison of HR net values. However, a test of interaction is most frequently recommended in methodology to compare two independent estimates of the same quantity derived from separate analyses (41). In the KEYNOTE-042 study, the effect remained significant in patients with a tumor proportion score (TPS) of 1% or greater, especially more than 50%. As we know, the expression of PD-L1 is not related to sex (42). Thus, the correlation between immune responses and sex has not been a consensus at present.

Biological differences between men and women could affect the susceptibility to certain respiratory diseases. Thus, the hypothesis on association between efficacy of immunotherapy and sex might be based on the following facts and several possible mechanisms may be involved. Estrogen plays an essential role in the immune system (43). Females show advantages in both innate and adaptive immune responses because of complex effects of X chromosome and sex hormones on the immune system and target organs (44). Thus, females have stronger immune environment than males (45). However, tumors in female patients exhibit stronger immune-suppressive signals (11). On the other hand, males provide an edge against females in some respects. As we know, TMB is an essential checkpoint before immunotherapy to predict efficacy of ICIs (42, 46). But evidence suggests that high-TMB is associated with smoking history, whereas common driver mutations in lung adenocarcinoma contributed to low-TMB (47). This conclusion implies that men with higher smoking frequency for gender dimorphism in behaviors may obtain greater benefit from ICIs. And the most sensitive populations to EGFR mutations are Asian females. Female patients may get higher mutation probability to have lower TMB and respond not well to immunotherapy (48).

To provide more powerful evidence, our study concentrated on NSCLC, one unitary type of cancer. To the best of our knowledge, this is the most comprehensive meta-analysis that has investigated this association in NSCLC patients up to now. We considered that it might make clear sense on clinical practice to research whether ICIs could have similar advantage over chemotherapy in different sex groups.

Referring to previous studies (11, 13), we made rigorous all-sided literature search strategies. We included 14 RCTs included in Wang’s study by literature review method. And we collected 7 new RCTs and updated 6 RCTs from 2018 to the present. Finally, we investigated OS data from 19 RCTs with 12037 patients and PFS data from 14 RCTs with 6940 patients. It is particularly noteworthy that subgroups from trials IMpower130 (28) and IMpower131 (30), demonstrated greater OS and PFS for females but not the same for males. They contributed crucially to the pooled HR effects. Differing from the results of the study done by Wang and colleagues (13), the key findings of this meta-analysis are that overall improvement of OS and PFS for both sexes patients in NSCLC of ICIs is evidence-supporting and there is no statistically significant association of patients’ sex with the efficacy of ICIs in NSCLC patients using both OS and PFS as outcomes. The subgroup analysis indicated that study methodology, pathological types, class of ICIs targets, and line of therapy were potential causes of between-study heterogeneity. Nonetheless, OS and PFS are regarded as gold standard and universally accepted benefit endpoint in oncology clinical trials (49). And there is no difference of OS and PFS between males and females in any subgroup, which we are more concerned with.

In addition, previous studies have only reported the association of patients’ sex with efficacy of ICIs in patients with NSCLC. Association between adverse effects and sex has not yet been defined. All trials included did not perform subgroups analysis of TRAEs among different sexes so that we could not acquire pooled estimates. Thus, we could not perform further exploration in the balance between efficacy of ICIs and following TRAEs. Incidence of TRAEs is one crucial safety outcome endpoint considered in clinical trials (50), and difference of TRAEs between males and females should have been paid more attention to.

Several potential limitations should be acknowledged for this meta-analysis. First, high degree of heterogeneity exists among articles in subgroup analysis. It may influence our analysis between different genders. Second, the sample size of females in all included trials was much smaller than that of males. This limitation can make statistical results more likely to be skewed towards males. Finally, some included studies lacked adequate data and we can’t acquire initial individual participants’ data from authors. Although the test of interaction helps us to compare the differences between male and female indirectly using HR and summary data, there is limited power to detect interactions. Analyses of individual data are needed to yield further insights.

In conclusion, *via* a comprehensive analysis of 21 articles, our findings indicated that NSCLC patients could achieve better OS and PFS from ICIs than chemotherapy regardless of their sex. Overall, the interaction between sex and immunotherapy efficacy is equal. Further investigations on the molecular mechanisms linking efficacy of ICIs to sex are also warranted.

## Data Availability Statement

The original contributions presented in the study are included in the article/**Supplementary Material**. Further inquiries can be directed to the corresponding author.

## Author Contributions

CX and SZ contributed equally to this work and are co-first authors. HC and SZ proposed the hypothesis and idea for the systematic review. All authors contributed to its development and the analysis plan. CX and HD did the literature search and reviewed studies for inclusion. XZ and JZ performed the data extraction and checking. CX, HD, and XL performed all meta-analyses and wrote the paper. All authors contributed to the article and approved the submitted version.

## Funding

This study was supported by Capital’s Funds for Health Improvement and Research (No. 2018-2-4065), National Natural Science Foundation of China (No. 81873396), and China-Japan Friendship Hospital.

## Conflict of Interest

The authors declare that the research was conducted in the absence of any commercial or financial relationships that could be construed as a potential conflict of interest.

## Publisher’s Note

All claims expressed in this article are solely those of the authors and do not necessarily represent those of their affiliated organizations, or those of the publisher, the editors and the reviewers. Any product that may be evaluated in this article, or claim that may be made by its manufacturer, is not guaranteed or endorsed by the publisher.

## Supplementary Material

Supplementary Figure 1 Funnel plots depicting subgroup analysis of OS data [**(A)** immune target; **(B)** line of therapy; **(C)** pathological type; **(D)** study methodology].

Supplementary Figure 2 Funnel plots depicting subgroup analysis of PFS data [**(A)** immune target; **(B)** line of therapy; **(C)** pathological type; **(D)** study methodology].

## Abbreviations

ICIs, Immune checkpoint inhibitors; OS, Overall survival; PFS, Progression-free survival; NSCLC, Non-small cell lung cancer; TRAEs, Treatment-related adverse events; RCTs, Randomized controlled trials; HR, Hazard ratio; 95% CI, 95% confidence interval; PROSPERO, Prospective register of systematic reviews; PRISMA, Preferred Reporting Items for Systematic Reviews and Meta-Analyses; MeSH, Medical subject headings; Patho, Pathological type; Chemo, Chemotherapy; IO, Immuno-oncology; NSCLC, Non-small cell lung cancer; PD-L1, Programmed cell death 1 ligand 1; PD-1, Programmed cell death protein 1; CTLA-4, Cytotoxic T - Lymphocyte Antigen 4; TMB, Tumor mutation burden, TPS, tumor proportion score.
