# Supplementary material for: Association Between Efficacy of Immune Checkpoint Inhibitors and Sex: An Updated Meta-Analysis on 21 Trials and 12,675 Non-Small Cell Lung Cancer Patients
Source: Front Oncol. 2021 Aug 26;11:627016. doi: 10.3389/fonc.2021.627016 (PMC8427763; doi:10.3389/fonc.2021.627016)
Supplement: Supplementary Figure 1 — Funnel plots depicting subgroup analysis of OS data [(A) immune target; (B) line of therapy; (C) pathological type; (D) study methodology]. [file DataSheet_1.docx]

Fig. S1 Funnel plots depicting subgroup analysis of OS data (A. immune target; B. line of therapy; C. pathological type; D. study methodology).


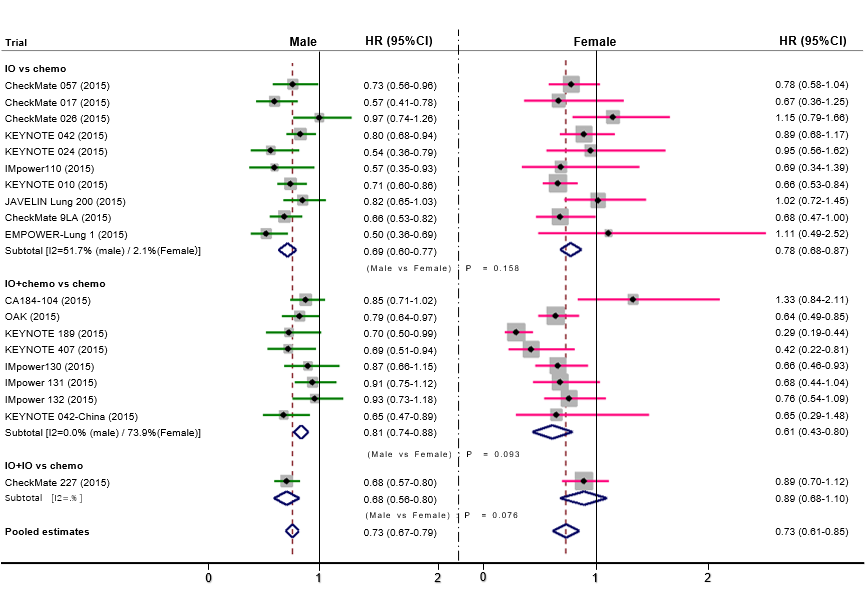

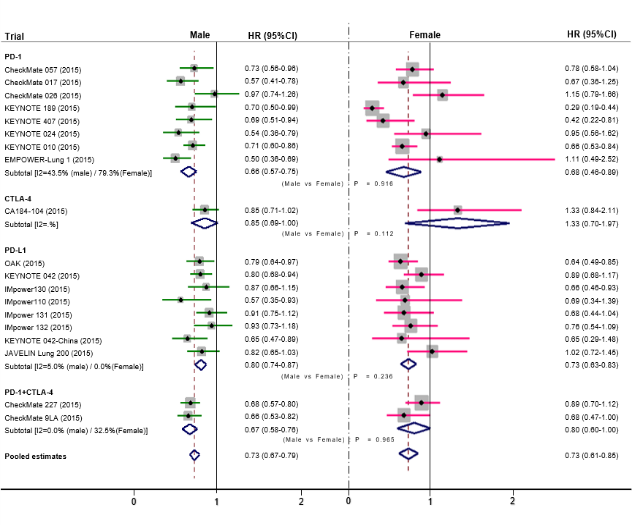

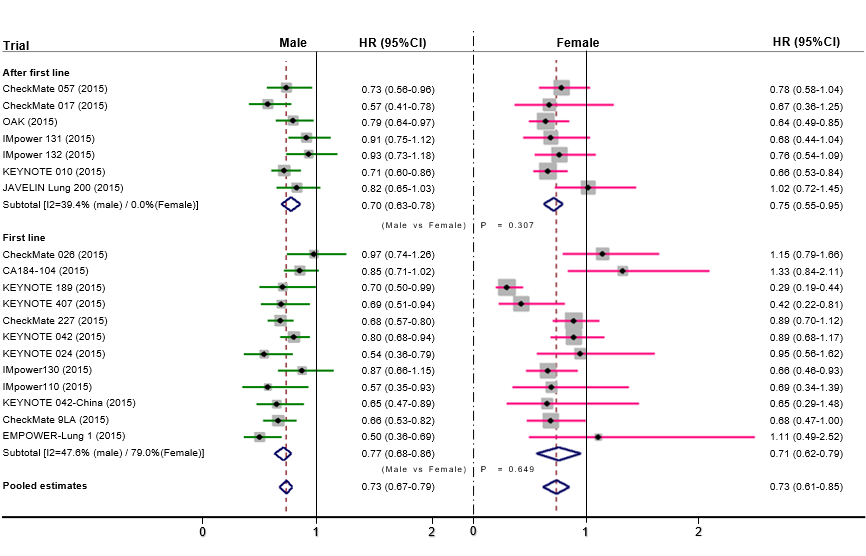

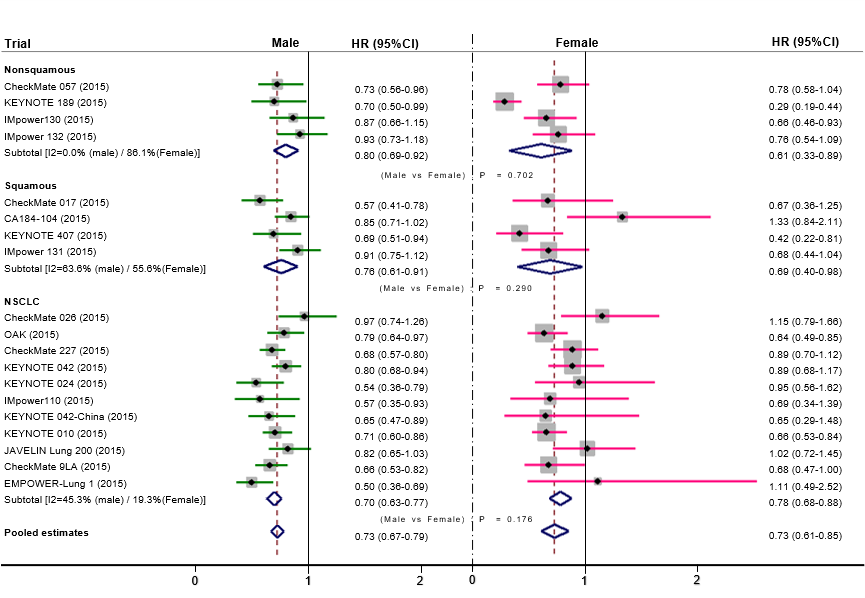


**A**

**D**

**C**

**B**

Fig. S2. Funnel plots depicting subgroup analysis of PFS data (A. immune target; B. line of therapy; C. pathological type; D. study methodology).


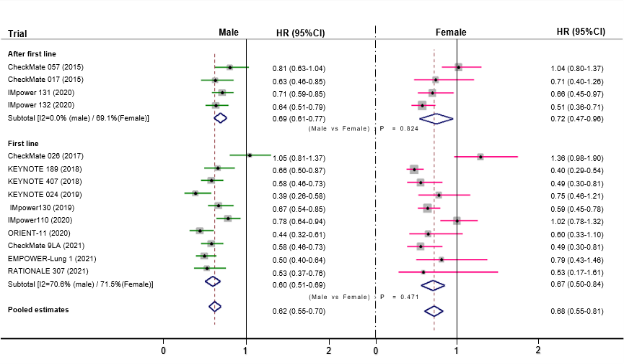

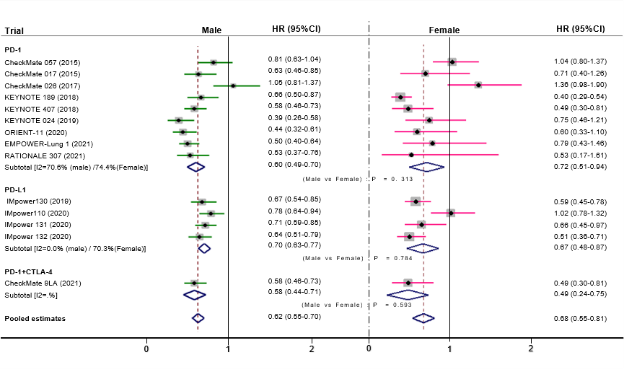

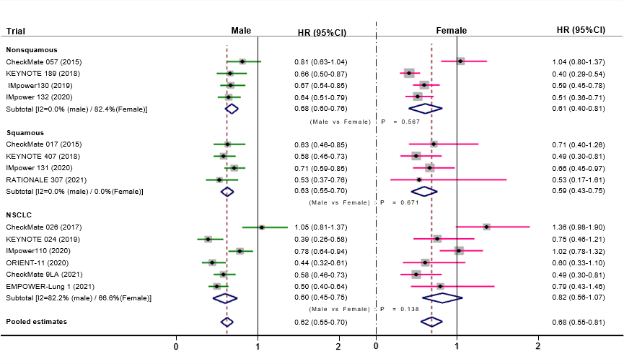

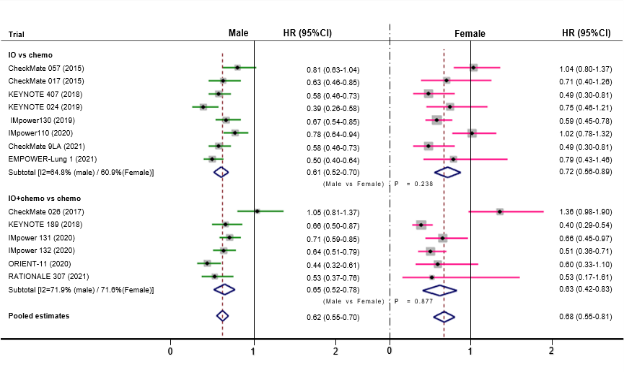


**A**

**B**

**C**

**D**
