# Supplementary material for: Association Between Efficacy of Immune Checkpoint Inhibitors and Sex: An Updated Meta-Analysis on 21 Trials and 12,675 Non-Small Cell Lung Cancer Patients
Source: Front Oncol. 2021 Aug 26;11:627016. doi: 10.3389/fonc.2021.627016 (PMC8427763; doi:10.3389/fonc.2021.627016)
Supplement: Supplementary file 2 [file Table_1.docx]

| **Identification** | **Line** | **Patho** | **Target** | **Study**  **methodology** | **Intervention (No.)** | **Control (No.)** | **Sex, (No.)** | | **Median follow-up, mo** |
| --- | --- | --- | --- | --- | --- | --- | --- | --- | --- |
|  |  |  |  |  |  |  | **male** | **female** |  |
| CheckMate 057 | >1 | Nonsquamous | PD‐1 | IO vs chemo | Nivolumab (292) | Chem (290) | 319 | 263 | >13.2 |
| CheckMate 017 | >1 | Squamous | PD‐1 | IO vs chemo | Nivolumab (135) | Chem (137) | 208 | 64 | >11 |
| CheckMate 026 | 1 | NSCLC | PD‐1 | IO+chemo vs chemo | Nivolumab (271) | Chem (270) | 332 | 209 | 13.50 |
| CA184‐104 | 1 | Squamous | CTLA‐4 | IO+IO vs chemo | Ipilimumab + Chem (388) | Chem (361) | 635 | 114 | Int: 12.5;Con: 11.8 |
| OAK | >1 | NSCLC | PD‐L1 | IO vs chemo | Atezolizumab (425) | Docetaxel (425) | 520 | 330 | 21.00 |
| KEYNOTE 189 | 1 | Nonsquamous | PD‐1 | IO+chemo vs chemo | Pembrolizumab + Chem (410) | Chem (206) | 363 | 253 | 10.50 |
| KEYNOTE 407 | 1 | Squamous | PD‐1 | IO vs chemo | Pembrolizumab + Chem (278) | Chem (281) | 455 | 104 | 7 .8 |
| CheckMate 227 | 1 | NSCLC | PD‐1 + CTLA‐4 | IO vs chemo | Nivolumab + Ipilimumab (583) | Chem (583) | 778 | 388 | >29.3 |
| KEYNOTE 042 | 1 | NSCLC | PD‐L1 | IO vs chemo | pembrolizumab(638) | Chem (637) | 902 | 372 | 12.8 (6.0–20.0) |
| KEYNOTE 024 | 1 | NSCLC | PD‐1 | IO vs chemo | Pembrolizumab (154) | Chem (151) | 187 | 118 | 25.20 |
| IMpower130 | 1 | Nonsquamous | PD‐L1 | IO vs chemo | Atezolizumab + Chem (451) | Chem (228) | 400 | 279 | Int:18.5(15.2–23.6); Con: 19.2(15.4–23.0) |
| IMpower110 | 1 | NSCLC | PD‐L1 | IO vs chemo | Atezolizumab (107) | Docetaxel (98) | 143 | 62 | 15.7 (0–35) |
| IMpower 131 | >1 | Squamous | PD‐L1 | IO+chemo vs chemo | Atezolizumab + Chem (343) | Chem (340) | 557 | 126 | 18.10 |
| IMpower 132 | >1 | Nonsquamous | PD‐L1 | IO+chemo vs chemo | Atezolizumab + Chem (292) | Chem (286) | 384 | 194 | 28.40 |
| KEYNOTE 042 (China) | 1 | NSCLC | PD‐L1 | IO vs chemo | pembrolizumab（128） | Chem（134） | 119 | 15 | 33.0 (25.6‒41.9） |
| ORIENT-11 | 1 | NSCLC | PD‐1 | IO+chemo vs chemo | sintilimab+chem (266) | Placebo+Chem (131) | 303 | 94 | 8.9 |
| KEYNOTE 010 | >1 | NSCLC | PD‐1 | IO+chemo vs chemo | Pembrolizumab (690) | Chem (343) | 634 | 399 | 67.4 (60.0-77.9) |
| JAVELIN Lung 200 | >1 | NSCLC | PD‐L1 | IO vs chemo | Avelumab (396) | Chem (396) | 367 | 162 | Int:34.7 (0.2–45.3);  Con:34.7 (0.03–44.4) |
| CheckMate 9LA | 1 | NSCLC | PD‐1 + CTLA‐4 | IO vs chemo | Nivolumab + Ipilimumab (361) | Chem (358) | 504 | 215 | 13.2 (6.4–17.0) |
| EMPOWER-Lung 1 | 1 | NSCLC | PD‐1 | IO vs chemo | Cemiplimab (283) | Chem (280) | 479 | 84 | 10.8 (7.6–15.8) |
| RATIONALE 307 | 1 | Squamous | PD‐1 | IO+chemo vs chemo | Tislelizumab + Chem (120) | Chem (121) | 218 | 23 | 8.6 (8.1-9.0) |

**Continued**

| **Identification** | **Overall Survival** |  |  | **PFS** |  |  |
| --- | --- | --- | --- | --- | --- | --- |
|  | **Overall HR (95% CI);  p value** | **HR (95% CI)  for men** | **HR (95% CI)  for women** | **PFS HR (95% CI);  p value** | **HR (95% CI)  for men** | **HR (95% CI)  for women** |
| CheckMate 057 | 0.73 (0.59-0.89); 0.002 | 0.73 (0.56‐0.96) | 0.78 (0.58‐1.04) | 0.92 (0.77–1.11); 0.39 | 0.81(0.63‐1.04) | 1.04 (0.80‐1.37) |
| CheckMate 017 | 0.59 (0.44-0.79); <0.001 | 0.57 (0.41‐0.78) | 0.67 (0.36‐1.25) | 0.62 (0.47 to 0.81; <0.001 | 0.63 (0.46‐0.85) | 0.71 (0.40‐1.26) |
| CheckMate 026 | 1.02 (0.80-1.30); NA | 0.97 (0.74‐1.26) | 1.15 (0.79‐1.66) | 1.15 (0.91-1.45); 0.25 | 1.05 (0.81‐1.37) | 1.36 (0.98‐1.90) |
| CA184‐104 | 0.91 (0.77-1.07); 0.25 | 0.85 (0.71‐1.02) | 1.33 (0.84‐2.11) | 0.87 (0.75-1.01); 0.07 | NA | NA |
| OAK | 0.73 (0.62–0.87); 0.0003 | 0.79 (0.64‐0.97) | 0.64 (0.49‐0.85) | NA | NA | NA |
| KEYNOTE 189 | 0.49 (0.38-0.64); <0.001 | 0.70 (0.50‐0.99) | 0.29 (0.19‐0.44) | 0.52 (0.43-0.64); <0.001 | 0.66 (0.50‐0.87) | 0.40 (0.29‐0.54) |
| KEYNOTE 407 | 0.64 (0.49-0.85); <0.001 | 0.69 (0.51‐0.94) | 0.42 (0.22‐0.81) | 0.56 (0.45-0.70); <0.001 | 0.58 (0.46–0.73) | 0.49 (0.30–0.81) |
| CheckMate 227 | 0.73 ( 0.64-0.84); NA | 0.68 ( 0.57-0.80) | 0.89 (0.70–1.12) | 0.79 (95% CI, 0.69–0.91); NA | NA | NA |
| KEYNOTE 042 | 0.81 (0.71–0.93); 0.0018 | 0.80 (0.68‐0.94) | 0.89 (0.68‐1.17) | 1.07 (0.94–1.21); NA | NA | NA |
| KEYNOTE 024 | 0.63 (0.47-0.86); NA | 0.54 (0.36‐0.79) | 0.95 (0.56‐1.62) | 0.50 (0.37–0.68); <0.001 | 0.39(0.26‐0.58) | 0.75(0.46‐1.21) |
| IMpower130 | 0.79 (0.64–0.98); 0.033 | 0.87 (0.66–1.15) | 0.66 (0.46–0.93) | 0.64 (0.54–0.77); <0.0001 | 0.67 (0.54–0.85) | 0.59 (0.45–0.78) |
| IMpower110 | 0.59 (0.40–0.89); 0.01 | 0.57 (0.35–0.93) | 0.69 (0.34–1.39) | 0.83(0.72-0.96); <0.005 | 0.78(0.64‐0.94) | 1.02(0.78‐1.32) |
| IMpower 131 | 0.88 (0.73–1.05); 0.16 | 0.91 (0.75-1.12) | 0.68 (0.44-1.04) | 0.71 (0.60–0.85); 0.0001 | 0.71 (0.59‐0.85) | 0.66 (0.45‐0.97) |
| IMpower 132 | 0.86 (0.71-1.06); 0.1546 | 0.93 (0.73-1.18) | 0.76 (0.54-1.09) | 0.60 (0.49, 0.72); <0.0001 | 0.64 (0.51‐0.79) | 0.51 (0.36‐0.71) |
| KEYNOTE 042 (China) | 0.67 (0.50-0.89); NA | 0.65 (0.47-0.89) | 0.65 (0.29-1.48） | 1.00 (0.76-1.31); NA | NA | NA |
| ORIENT-11 | 0.61 (0.40-0.93); 0.01921 | NA | NA | 0.48 (0.36-0.64); <0.00001 | 0.44 (0.32-0.61) | 0.60 (0.33,1.10) |
| KEYNOTE 010 | 0.70 (0.61 ‒0.80); NA | 0.71 (0.60 ‒0.86) | 0.66 (0.53 ‒0.84) | 0.84 (0.73–0.96); NA | NA | NA |
| JAVELIN Lung 200 | 0.90 (0.72–1.12); 0.16 | 0.82 (0.65–1.03) | 1.02 (0.72–1.45) | NA | NA | NA |
| CheckMate 9LA | 0.66 (0.55–0.80); NA | 0.66 (0.53–0.82) | 0.68 (0.47–1.00) | 0.56(0.45-0.70); <0.001 | 0.58 (0.46–0.73) | 0.49 (0.30–0.81) |
| EMPOWER-Lung 1 | 0.57 (0.42–0.77); 0.0002 | 0.50 (0.36–0.69) | 1.11 (0.49–2.52) | 0.54 (0.43–0.68); <0.0001 | 0.50 (0.40–0.64) | 0.79 (0.43–1.46) |
| RATIONALE 307 | NA | NA | NA | 0.52 (0.37-0.74); <0.001 | 0.53 (0.37-0.76) | 0.53 (0.17-1.61) |

Tab. S1. Details of included trials

Abbreviation: Inv=Intervention group; Con=Control group; Patho=Pathological type; Chemo=Chemotherapy; IO=Immunotherapy; NSCLC=Non-small cell lung cancer; PD-L1= Programmed cell death 1 ligand 1; PD-1= Programmed cell death protein 1; CTLA-4= Cytotoxic T - Lymphocyte Antigen 4; HR=Hazard ratio; OS=Overall survival; PFS=Progression free survival；NA=Not available
