# Supplementary material for: Association Between Efficacy of Immune Checkpoint Inhibitors and Sex: An Updated Meta-Analysis on 21 Trials and 12,675 Non-Small Cell Lung Cancer Patients
Source: Front Oncol. 2021 Aug 26;11:627016. doi: 10.3389/fonc.2021.627016 (PMC8427763; doi:10.3389/fonc.2021.627016)
Supplement: Supplementary file 3 [file Table_2.docx]

The modified Jadad scoring system for RCTs (from Crowther Met al.Blood.

2010^[[1]](#endnote-1)^):

Question 1.Was the study described as randomized? lf yes, score 1 point.

Question 2. lf yes to question 1, was an appropriate randomization sequence described and used

(eg, table of random numbers,

computer generated, etc.)? lf yes, score 1 point.

Question 3. lf yes to question 1, was an inappropriate method to generate the sequence of randomization used (patients were allocatedalternately, or according to date of birth, hospital number, etc.)? lf yes, subtract 1 point.

Question 4. Was the study described as double blinded? lf yes, score 1 point.

Question 5. lf yes to question 4, was an appropriate method of blinding used (eg, identical placebo, active placebo, dummy, etc.)2 f yes,score 1 point.

Question 6. lf yes to question 4, was an inappropriate method for blinding used (eg, comparison of tablet vs injection with no doubledummy)? lf yes, subtract 1 point.

Question 7.Were the withdrawals and dropouts described? lf yes, score 1 point.

|  |  |  |  |  |  |  |  |  |
| --- | --- | --- | --- | --- | --- | --- | --- | --- |
| Author (year) | Question 1 | Question 2 | Question 3 | Question 4 | Question 5 | Question 6 | Question 7 | Score |
| Borghaei 2015 | Yes | No | No | No | No | No | Yes | 2 |
| Brahmer 2015 | Yes | No | No | No | No | No | Yes | 2 |
| Carbone 2017 | Yes | No | No | No | No | No | Yes | 2 |
| Gandhi 2018 | Yes | Yes | No | Yes | Yes | NO | Yes | 5 |
| Govindan 2017 | Yes | Yes | No | Yes | Yes | No | Yes | 5 |
| Hellmann 2019 | Yes | No | No | No | No | No | Yes | 2 |
| Herbst 2020 | Yes | Yes | No | No | No | No | Yes | 3 |
| Jotte 2020 | Yes | No | No | No | No | No | Yes | 2 |
| Mok 2019 | Yes | Yes | No | No | No | No | Yes | 3 |
| Nishio 2020 | Yes | Yes | No | No | No | No | Yes | 3 |
| Paz‐Ares 2018 | Yes | Yes | No | Yes | Yes | No | Yes | 5 |
| Reck 2019 | Yes | No | No | No | No | No | Yes | 2 |
| Rittmeyer 2017 | Yes | Yes | No | No | No | No | Yes | 3 |
| Wu 2020 | Yes | No | No | No | No | No | Yes | 2 |
| West 2019 | Yes | Yes | No | No | No | No | Yes | 3 |
| Yang 2020 | Yes | Yes | No | No | No | No | Yes | 3 |
| Herbst  2021 | Yes | Yes | No | No | No | No | Yes | 3 |
| Park  2021 | Yes | Yes | No | No | No | No | Yes | 3 |
| Paz-Ares  2021 | Yes | Yes | No | No | No | No | Yes | 3 |
| Sezer  2021 | Yes | Yes | No | No | No | No | Yes | 3 |
| Wang  2021 | Yes | Yes | No | No | No | No | Yes | 3 |

1. Crowther M, Lim W, Crowther MA. Systematic review and meta-analysis methodology. Blood. 2010 Oct 28;116(17):3140-6. [↑](#endnote-ref-1)
